# Tertiary lymphoid structures in thyroid cancer

**DOI:** 10.20945/2359-4292-2025-0103

**Published:** 2025-09-22

**Authors:** Katia Sakimi Nakadaira, Kelly Cristina Saito, Cesar Seigi Fuziwara, Patricia Künzle Ribeiro Magalhães, Leandra Naira Zambelli Ramalho, Julio C. Ricarte-Filho, Lea Maria Zanini Maciel, Edna Teruko Kimura

**Affiliations:** 1 Departamento de Biologia Celular e do Desenvolvimento, Instituto de Ciências Biomédicas, Universidade de São Paulo, São Paulo, SP, Brasil; 2 Divisão de Endocrinologia, Departamento de Clínica Médica, Faculdade de Medicina de Ribeirão Preto, Universidade de São Paulo, Ribeirão Preto, SP, Brasil; 3 Departamento de Patologia e Medicina Legal, Faculdade de Medicina de Ribeirão Preto, Universidade de São Paulo, Ribeirão Preto, SP, Brasil; 4 Division of Endocrinology and Diabetes, Children’s Hospital of Philadelphia, University of Pennsylvania, Philadelphia, PA, USA

**Keywords:** Papillary thyroid carcinoma, tertiary lymphoid structure maturation, tumor-infiltrating B cells, morphological analysis, lymphoid neogenesis

## Abstract

**Objective:**

This study aimed to investigate the presence of tertiary lymphoid structures (TLSs) and tumor-infiltrating B cells within the germinal centers of TLSs in the tumor microenvironment of thyroid cancer, utilizing a morphological approach.

**Materials and methods:**

Histological samples from patients with papillary thyroid carcinoma (PTC) (n = 112) stained with hematoxylin and eosin were examined. The presence of lymphoid neogenesis in PTC was determined based on morphological features and classified according to TLS location and maturation status. Immunofluorescence staining was performed on selected cases to identify B cells within mature TLSs. Additionally, 499 scanned slides from the PTC cohort in The Cancer Genome Atlas - Thyroid Carcinoma (TCGA-THCA) dataset were accessed via cBioPortal to assess the presence of TLSs and compare the clinical and molecular characteristics of PTC cases with and without TLSs.

**Results:**

Tertiary lymphoid structures, resembling ectopic lymph nodes, were identified in 41% (46/112) of the histological PTC samples. Among these, 63% (29/46) were located in peritumoral regions, while 13% (6/46) were found within the intratumoral area. Mature TLSs containing germinal centers, in which B cells were detected, were observed in 15% (7/46) of cases. Immature TLSs were detected in 52% (24/46) of PTC cases with TLSs. Analysis of PTC scanned images from cBioPortal revealed TLSs in 8.4% of cases, of which 62% harbored the BRAFV600E mutation, along with upregulation of immune cell markers and *SLC5A5* (NIS) expression.

**Conclusion:**

The identification of TLSs across multiple malignancies underscores their functional significance in modulating tumor-immune interactions with clinical implications. Therefore, the identification and morphological characterization of TLSs in PTC may provide valuable insights into their potential as immunobiomarkers in thyroid cancer.

## INTRODUCTION

Thyroid cancer is the most common endocrine malignancy, with papillary thyroid carcinoma (PTC) accounting for approximately 84% of all cases (^1^,^2^). The thyroid gland, an endocrine organ, shows signs of immune activity, as evident in chronic lymphocytic thyroiditis, which is characterized by a dense inflammatory infiltrate of lymphocytes (^3^). Similarly, tumor-infiltrating leukocytes are often observed within the tumor microenvironments of differentiated thyroid carcinomas (^4^).

The complex network comprising malignant cells, stromal cells, and tumor-infiltrating immune cells within the tumor microenvironment has been extensively studied (^5^). Among these immune components, tertiary lymphoid structures (TLSs) within various cancer types’ microenvironments have been the focus of recent studies (^6^-^8^). Tertiary lymphoid structures, indicative of lymphoid neogenesis, display varying degrees of maturation and are located either within the tumor parenchyma or the peritumoral area, often at the invasive margin (^9^,^10^). The peritumoral location of TLSs within the tumor microenvironment is considered a key site for an anti-tumor immune response. Immature TLSs are small lymphoid aggregates that evolve into larger, rounded structures. Mature TLSs (m-TLSs) are characterized by organized structures with germinal centers (GCs), which are oval, clear areas enriched with B cells at the center of the TLSs (^11^). Morphologically, TLSs is typically assessed by hematoxylin and eosin staining on formalin-fixed paraffin-embedded tumor samples (^12^).

In thyroid tissue, the presence of infiltrating leukocytes and TLSs is commonly associated with autoimmune diseases, especially Hashimoto’s thyroiditis (^3^,^12^). However, the detailed morpho-logical characterization of TLSs within the tumor microenvironment remains underexplored. Given the mounting evidence supporting the significance of B cell-rich TLSs in various cancers, this study aims to explore the presence, localization, maturation status, and prognostic relevance of TLSs in PTC. These findings may contribute to identifying novel biomarkers and enhance the understanding of tumor immunology in thyroid cancer.

## MATERIALS AND METHODS

Papillary thyroid carcinoma samples were obtained from the archival of the Department of Pathology at Ribeirão Preto Medical School and the Department of Cell and Developmental Biology at the Institute of Biomedical Sciences at the University of São Paulo. The study was carried out in accordance with the guidelines of the Human Ethics Committee of the Institute of Biomedical Science (CAAE no. 65317522.3.00000.5467).

The 112 hematoxylin and eosin-stained slides of PTC were examined under light microscopy for TLSs detection and classification based on their localization and maturation (^10^). For selected cases, the immunofluorescence assay was conducted to identify B cells within TLSs. The slides were incubated with a polyclonal rabbit antibody against CD 20 (PA5-16701, Thermo Fisher Scientific, USA), followed by a secondary goat anti-rabbit antibody conjugated with Alexa Fluor^®^ 488 (A11008, Thermo Fisher Scientific, USA). Fluorescence images were captured using a Nikon Eclipse E600 microscope. Additionally, 499 PTC images of hematoxylin and eosin-stained histological samples from The Cancer Genome Atlas - Thyroid Carcinoma (TCGA-THCA) dataset, accessed from cBioPortal (https://www.cBioPortal.org), were analyzed to study TLS morphology (^13^,^14^).

**Supplementary Figure 1:**
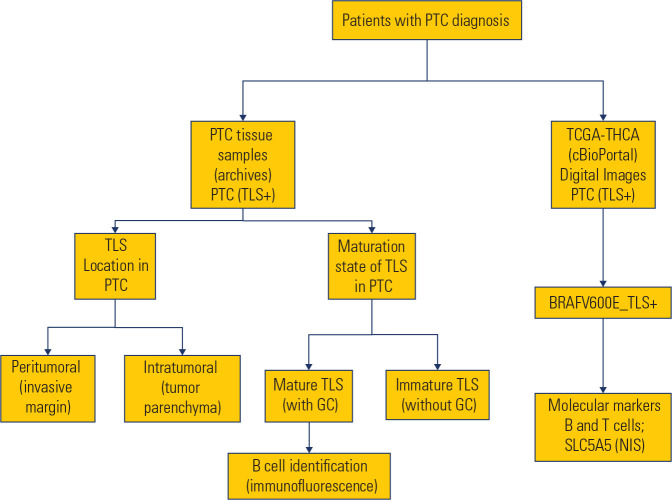
Flowchart of the study of Tertiary Lymphoid Structures (TLSs) in Papillary Thyroid Carcinoma. PTC: papillary thyroid carcinoma. **TLS:** tertiary lymphoid structures. **TCGA:** The Cancer Genome Atlas. **THCA:** Thyroid Carcinoma. **GC:** germinal center. **BRAFV600E:** BRAF mutated protein. ***SLC5A5* (NIS):** Solute Carrier Family 5 Member 5 gene (Sodium/Iodide Symporter).

The PTC cases with detected TLS were stratified according to BRAF and RAS mutation status. The expression of molecular markers, including B and T cell markers and *SLC5A5* (NIS), was evaluated among patients with both TLSs and BRAF mutations. Statistical analysis was conducted using GraphPad Prism (v 5.00), considering *p* < 0.05 as statistically significant. Categorical variables were analyzed using the chi-square test. Survival analysis employed the Kaplan-Meier method, with differences between survival curves evaluated using the Log-Rank test. The workflow diagram (**Supplementary Figure 1**) provides an overview of the PTC patient cohort included in this study.

## RESULTS

**Figure 1:**
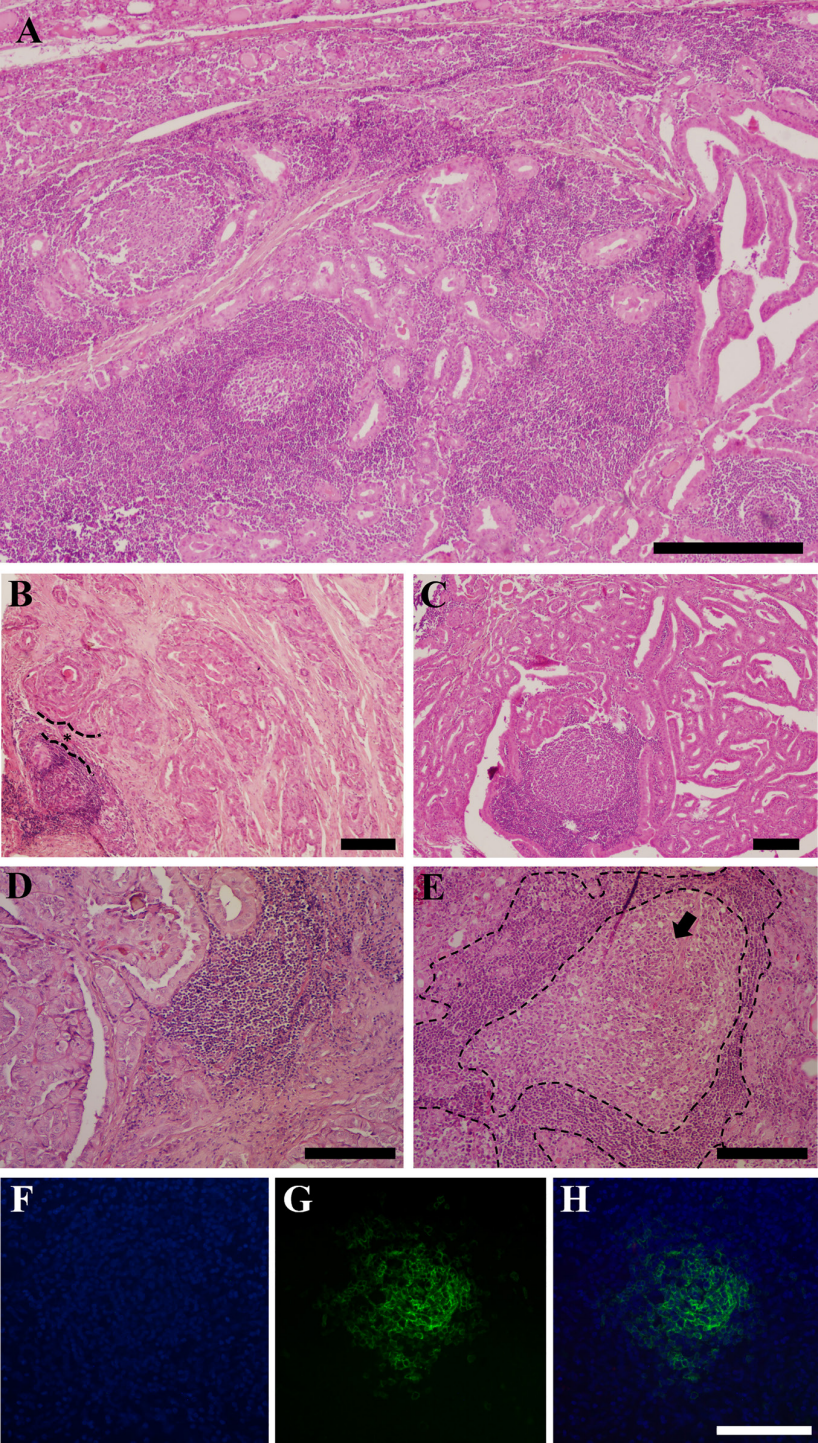
Tertiary lymphoid structures (TLSs) associated with papillary thyroid carcinoma (PTC): photomicrography of TLSs detected in formalin-fixed paraffin-embedded tumor sections stained by hematoxylin and eosin (A-E) and by immunofluorescence assay (F-H). **A**) Low-power view of TLSs distributed in the tumor microenvironment of PTC. A dense population of small lymphocytes form nodular aggregates. **B**) Peritumoral location of the TLS. Lymphoid neogenesis close to the infiltrative border of papillary thyroid carcinoma. *The proximity of TLS and papillary architecture is illustrated. **C**) Intratumoral location of the TLS. The nodular structure is composed of lymphoid cells in the parenchyma of the tumor, surrounded by papillary structures. **D**) Immature-TLS. High-power view showing diffuse monotonous lymphocytes infiltrate without clear germinal centers (GC). Note the juxtaposition of papillary architecture (left) and immature TLS. **E**) Mature TLSs. High-power view exhibiting lymphoid aggregate with prominent round and clear GC zone (arrow). **F**) DAPI, nuclei stained in blue. **G**) Immunofluorescence staining of TLS with GC in papillary thyroid carcinoma. Identification of CD20+ stained in green, characterizing the presence of B cells in the GC. **H**) Merged images (anti-CD20 and DAPI). Image scale bar: A = 400 µm, B = 200 µm, and F-H = 100 µm.

One hundred and twelve tissue specimens from patients with PTC were screened for TLSs presence, which were identified in 46 of the 112 cases (41.0%) within the PTC microenvironment (**Figure 1A**). Histo-logical assessment revealed variations in the location and maturation stages of TLSs. These structures were predominantly identified in the peritumoral area (29/46 cases, 63% **Figure 1B**), while a smaller proportion were found in the intratumoral site (6/46, 13%; **Figure 1C**). Concurrent peritumoral and intratumoral TLS appeared in 11 out of 46 cases (24%).

The maturation of TLSs is characterized by the absence or presence of an organized structure rich in B lymphocytes within the central core, known as the GC. Immature TLSs, lacking a GC, were identified in 24 out of 46 (52%) of PTC cases, while mature morphology, with the presence of a GC, was identified in 7 out of 46 (15%) cases (**Figures 1D and 1E**). Both mature and immature TLSs were detected in the same sample in 15 out of 46 cases (33%).

**Supplementary Figure 2:**
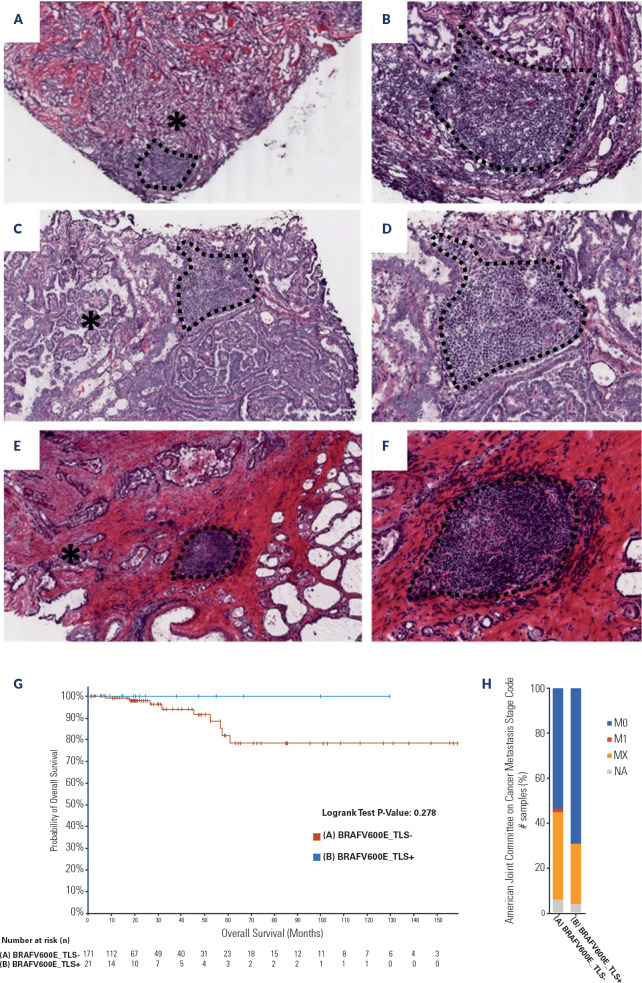
TCGA Thyroid Carcinoma (THCA) digital histological samples obtained from cBioPortal and clinical prognosis. A and **B:** TLS in lower power view of (A) and medium power view (B) in the tumor microenvironment. **C** and **D:** TLS within the papillary architecture and parenchyma of PTC. Dense population of small lymphocytes forming nodular structure, lower (C) and higher magnification (D). **E and F:** TLS Lymphoid aggregates in peritumoral area in the stroma, low (E) and high (F) magnification. A, C and E - PTC (*). **G** and **H:** Clinical significance of TLSs in PTC patients with BRAFV600E. BRAF_TLS- (n=210) vs BRAFV600E_TLS+ (n=26) in TCGA Thyroid Cancer (THCA) - cBioPortal. G: Kaplan-Meier survival curve of probability of overall survival between BRAFV600E_TLS+ group and BRAFV600E_TLS- group. H: Comparison of cancer stage metastasis between patients with PTC, BRAFV600E_TLS+ group and BRAF_TLS-. **M0:** cancer has not spread to other parts of the body; **M1:** Cancer has spread to other parts of the body; **MX:** Metastasis can not be measured; **NA:** not available.

Immunofluorescence staining confirmed the pre-sence of B cells within GCs of TLSs in selected cases (**Figures 1F and 1H**). Analysis of digital images from 499 thyroid carcinoma patients in the TCGA-THCA dataset revealed the presence of TLSs in 8.4% of cases (42/499; **Supplementary Figure 2**). **Table 1** summarizes the clinico-pathological and molecular features of the TCGA cohort. The TLS-positive PTCs were predominantly associated with the BRAFV600E mutation (26 out of 42; 62%), while all 52 H-K-NRAS-mutant PTCs were TLS-negative. RAS mutations were observed in 11.4% of TLS-negative PTCs and in 10.4% of the overall PTC cohort.

**Table 1:** Distribution of clinical characteristics of TCGA - PTC cohort with or without TLSs

Clinical Characteristics	Variable	TLS+ n (%)	TLS- n (%)	Chi-square Test p-value
Gender	Male	12 (28.6)	122 (26.7)	0.793
	Female	30 (71.4)	335 (73.3)	
Age	≤ 55	31 (73.8)	320 (70.0)	0.607
	> 55	11 (26.2)	137 (30.0)	
PTC subtype	Classic	31 (73.8)	323 (70.7)	0.368
	Tall cell	6 (14.3)	97 (21.2)	
	Follicular	5 (11.9)	30 (6.6)	
	Other	0 (0)	7 (1.5)	
BRAF mutation status	BRAFV600E	26 (61.9)	257 (56.2)	0.478
	BRAF WT	16 (38.1)	200 (43.8)	
RAS mutation status	N/H/KRAS	0 (0)	59 (12.9)	0.013
	N/H/KRAS WT	42 (100)	398 (87.1)	
AJCC Neoplasm disease stage	Stage I	23 (54.8)	259 (56.7)	0.064
	Stage II	0 (0)	51 (11.2)	
	Stage III	15 (35.7)	95 (20.8)	
	Stage IV	0 (0)	2 (0.4)	
	Stage IVA	3 (7.1)	42 (9.2)	
	Stage IVC	0 (0)	6 (1.3)	
	NA	1 (2.4)	2 (0.4)	
Tumor stage	pT1	6 (14.3)	37 (8.1)	0.468
	pT1A	3 (7.1)	17 (3.7)	
	pT1B	9 (21.4)	71 (15.5)	
	pT2	11 (26.2)	155 (33.9)	
	pT3	13 (31.0)	153 (33.5)	
	pT4	0 (0)	8 (1.8)	
	pT4A	0 (0)	14 (3.1)	
	pTX	0 (0)	2 (0.4)	
Lymph node metastasis	pN0	19 (45.2)	208 (45.5)	0.118
	pN1	3 (7.1)	55 (12.0)	
	pN1A	13 (31.0)	79 (17.3)	
	pN1B	6 (14.3)	66 (14.4)	
	pNx	1 (2.4)	49 (10.7)	
Distant metastasis	M0	29 (69.0)	248 (54.3)	0.276
	M1	0 (0)	9 (2.0)	
	MX	13 (31.0)	199 (43.5)	
	NA	0 (0)	1 (0.2)	
Disease free status	Disease free	30 (71.4)	299 (65.4)	0.722
	Recurred/progressed	2 (4.8)	23 (5.0)	
	NA	10 (23.8)	135 (29.5)	

**Supplementary Figure 3:**
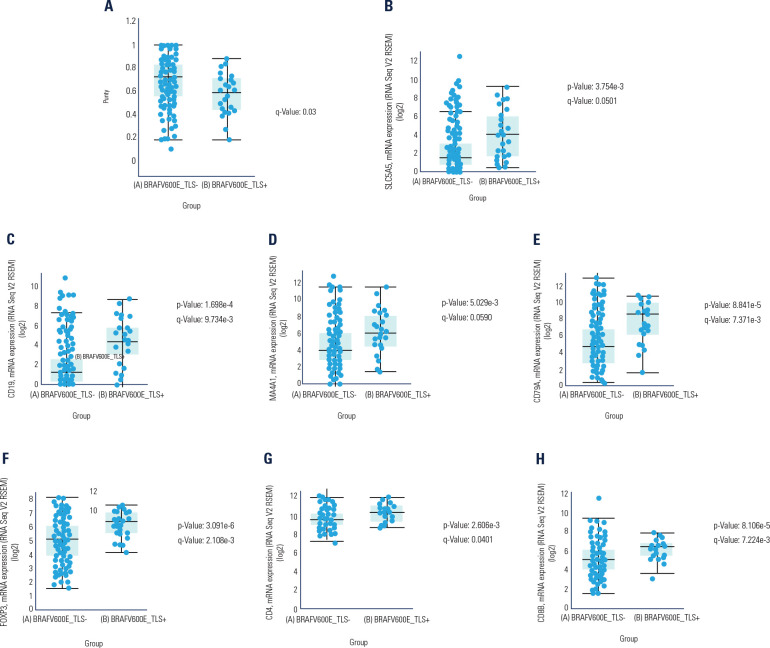
Comparison of Molecular Marker Expression Between BRAFV600E PTCs with and without TLSs. A: Tumor purity. **B:** *SLC5A5* (NIS). B cells markers (C, D, E): **C:** *CD19*. **D:** *CD20/MS4A1*. **E** - *CD79A*. T cells markers (F, G, H): **F:** *FOXP3* (regulatory T cells), **G:** *CD4* (helper T cells). **H:** *CD8B* (cytotoxic T cells). Data were obtained from the PTC cohort of The Cancer Genome Atlas - Thyroid Carcinoma (TCGA-THCA), and the graphic was generated using the public dataset available at cBioPortal (www.cbioportal.org, accessed September 2024).

Given the high prevalence of BRAFV600E mutations in TLSs-positive PTCs, we compared BRAFV600E-mutant PTC cases with (+) and without (^11^,^16^,^17^) TLSs to identify differences in their clinical and molecular features. Kaplan-Meier survival analysis showed a trend toward improved survival in the BRAFV600E TLSs-positive group, although the difference was not statistically significant (**Supplementary Figure 2G**). In addition, TLSs-positive BRAFV600E PTCs exhibited reduced tumor purity, which is consistent with increased immune infiltration in these tumors. In fact, several TLSs-related and immune cell markers were upregulated in BRAFV600E TLS-positive PTCs, including B cell markers (*CD19, CD20/MS4A1*, and *CD79A*) and T cell markers (*FOXP3, CD4*, and *CD8A/CD8B*) (^15^-^17^). Similarly, *SLC5A5* expression, which encodes the sodium/iodide symporter (NIS), was significantly elevated in the BRAFV600E TLS-positive group (**Supplementary Figure 3**).

## DISCUSSION

This study investigated TLSs in the PTC tissue samples. The morphological characteristics of TLSs observed in PTC are consistent with those found in other types of tumors. Tertiary lymphoid structures have been widely reported in the tumor microenvironments of various cancers, including pancreatic, colorectal, and lung cancers (^8^,^10^). Similar to findings in other cancers, most TLSs in our PTC samples were located in the peritumoral area, rather than within the tumor parenchyma (^11^).

Depending on their maturation state, TLSs exhibi-ted stages of maturation, displaying morphological heterogeneity in the tumor microenvironment. TLSs are composed primarily of T and B cells, which expand and organize into distinct compartments, resembling a lymph node. The m-TLSs are characterized by the presence of GCs, which are B cell-rich areas within the TLSs core (^18^).

In this study, B cells within the GCs of m-TLSs in PTC tissue were identified using an immunofluorescence approach, confirming the identity of m-TLSs. T cells are traditionally considered the primary mediators of anti-tumor immunity, leading to the development of immune checkpoint therapies. However, these treatments, designed to enhance T cell activity, do not benefit many patients, often due to T cell dysfunction and tumor immune escape mechanisms (^19^).

In this context, B cells within the GCs of m-TLSs are increasingly recognized for their crucial role in effective anti-tumor immune responses and as potential biomarkers for the efficacy of immunotherapy (^7^,^9^). B cells contribute through various mechanisms, including the production of reactive antibodies, secretion of pro-inflammatory cytokines, antigen presentation to intratumoral T cells, and direct tumor lysis (^20^). Li and cols. (^21^-^23^) recently suggested that B cells exert anti-tumor effects in PTC through the formation of TLSs, which are associated with improved patient prognosis.

In our study, the detection rate of TLSs in formalin-fixed paraffin-embedded archival samples was 41.9%, whereas the analysis of TCGA PTC scanned slides showed a significantly lower frequency (8.4%). This discrepancy may be attributed to the limitation that only one scanned slide per patient was available on the cBioPortal platform, possibly not representing the entire tumor. Additionally, the available images primarily depict the tumor parenchyma, excluding the invasive margins and surrounding tissue areas where TLSs are more commonly found.

The clinicopathological characteristics of 42 PTC patients with TLSs detected in TCGA images revealed that 62% harbored the BRAFV600E mutation (**Table 1**) and showed a trend toward improved overall survival according to the Kaplan-Meier method (**Supplementary Figure 1**). The BRAF mutation is the most prevalent genetic alteration in PTC, yet the association between the BRAFV600E mutation and more aggressive PTC remains controversial (^1^,^24^-^26^).

In this context, BRAFV600E TLS-positive cases may be associated with a better prognosis compared to those without TLSs, especially when considering distant metastasis (M stage) (Supplementary **Figure 2H**). Furthermore, *SLC5A5* (NIS) expression was significantly higher in TLS-positive PTCs among BRAF-mutant cases. The loss of cellular differentiation in thyroid carcinomas is often linked with reduced or absent NIS expression, which contributes to the failure of radioiodine therapy and the development of treatment-refractory disease (^7^,^27^,^28^).

The growing evidence of TLS detection in various cancers with favorable prognoses underscores their potential as prognostic biomarkers (^20^). The association of TLS with clinical parameters in thyroid cancer is still emerging (^5^), and further studies can provide new insights into the role of TLS-positive tumor microenvironments in thyroid tumorigenesis. This study offers a morphological characterization of TLSs in histological samples of PTC, presenting an easily accessible and cost-effective approach that could be added as a valuable parameter for guiding clinical management in thyroid cancer in the future.

## Data Availability

datasets related to this article will be available upon request to the corresponding author.
